# Waterlogging of Winter Crops at Early and Late Stages: Impacts on Leaf Physiology, Growth and Yield

**DOI:** 10.3389/fpls.2018.01863

**Published:** 2018-12-20

**Authors:** Rocío Antonella Ploschuk, Daniel Julio Miralles, Timothy David Colmer, Edmundo Leonardo Ploschuk, Gustavo Gabriel Striker

**Affiliations:** ^1^IFEVA, Facultad de Agronomía, Universidad de Buenos Aires, CONICET, Buenos Aires, Argentina; ^2^Faculty of Science, School of Agriculture and Environment, The University of Western Australia, Crawley, WA, Australia; ^3^Facultad de Agronomía, Cátedra de Cultivos Industriales, Universidad de Buenos Aires, Buenos Aires, Argentina

**Keywords:** waterlogging, crops, aerenchyma, photosynthesis, yield

## Abstract

Waterlogging is expected to increase as a consequence of global climate change, constraining crop production in various parts of the world. This study assessed tolerance to 14-days of early- or late-stage waterlogging of the major winter crops wheat, barley, rapeseed and field pea. Aerenchyma formation in adventitious roots, leaf physiological parameters (net photosynthesis, stomatal and mesophyll conductances, chlorophyll fluorescence), shoot and root growth during and after waterlogging, and seed production were evaluated. Wheat produced adventitious roots with 20–22% of aerenchyma, photosynthesis was maintained during waterlogging, and seed production was 86 and 71% of controls for early- and late-waterlogging events. In barley and rapeseed, plants were less affected by early- than by late-waterlogging. Barley adventitious roots contained 19% aerenchyma, whereas rapeseed did not form aerenchyma. In barley, photosynthesis was reduced during early-waterlogging mainly by stomatal limitations, and by non-stomatal constraints (lower mesophyll conductance and damage to photosynthetic apparatus as revealed by chlorophyll fluorescence) during late-waterlogging. In rapeseed, photosynthesis was mostly reduced by non-stomatal limitations during early- and late-waterlogging, which also impacted shoot and root growth. Early-waterlogged plants of both barley and rapeseed were able to recover in growth upon drainage, and seed production reached *ca*. 79–85% of the controls, while late-waterlogged plants only attained 26–32% in seed production. Field pea showed no ability to develop root aerenchyma when waterlogged, and its photosynthesis (and stomatal and mesophyll conductances) was rapidly decreased by the stress. Consequently, waterlogging drastically reduced field pea seed production to 6% of controls both at early- and late-stages with plants being unable to resume growth upon drainage. In conclusion, wheat generates a set of adaptive responses to withstand 14 days of waterlogging, barley and rapeseed can still produce significant yield if transiently waterlogged during early plant stages but are more adversely impacted at the late stage, and field pea is not suitable for areas prone to waterlogging events of 14 days at either growth stage.

## Introduction

Over the last decades, the number of waterlogging episodes on croplands has increased worldwide, mainly due to more intense and unpredictable rainfalls associated with climate change (Hirabayashi et al., 2013; Core Writing Team et al., 2014). In turn, soils with significant content of clay or intensely compacted by the repeated use of agricultural machinery can experience poor drainage, also entailing an increase in waterlogging occurrence (Jackson, 2004). Waterlogging impacts around 16% of soils in United States, affecting also irrigated areas of India, China, and Pakistan (Pang et al., 2004). Moreover, the occurrence of flash floods is expected to increase in Europe due to an intensified hydrological cycle by global warming (Feyen et al., 2012). In the Argentine Pampas, a vast plain of around 50 Mha, about 31% of the area has suffered recurrently from waterlogging events since 2002 (Kuppel et al., 2015). To illustrate economic losses associated with waterlogging or floods, in the United States the lost crop production was around $360 million per year during 2010–2016, and was even a greater loss than caused by drought in three out of the 7 years (Pedersen et al., 2017; USDA, 2017). So, soil waterlogging is a major abiotic stress of increasing importance, and it causes significant yield losses of various crops. Therefore, it is necessary to understand how crop plants respond to waterlogging to identify traits contributing to tolerance.

Wheat and barley are the most substantial cool-season cereals in the world, each having an annual production, respectively, of up to 749 and 141 million tons FAOSTAT, 2016. Rapeseed is the second largest oil-seed crop after soybean, presenting a production of 68 million ton FAOSTAT, 2016, providing oil with a high level of omega-9 (oleic fatty acid), one of the healthiest oils (Kwon et al., 1991). Field pea is an important cool season grain legume, with about 14 million ton of dry peas produced each year FAOSTAT, 2016. Each of these four species can suffer yield losses resulting from waterlogging (Siddique et al., 1993; Zhou and Lin, 1995; Setter and Waters, 2003; Pang et al., 2004). Depending on the waterlogging duration, soil type and genotypes, reductions in yield range from 15 to 25% in wheat and barley (Setter and Waters, 2003; Herzog et al., 2016), 30–45% in rapeseed (Gutierrez Boem et al., 1996), and 50–90% in field pea (Cannell et al., 1979).

Soil waterlogging imposes a considerable slow-down of the oxygen exchange between soil and roots, as gas diffusion rates are 10,000 times slower in water than in air (Armstrong, 1979). Consequently, waterlogged soils can quickly become anoxic at depths greater than a few centimeters, as the oxygen demand by roots and soil microorganisms’ respiration largely exceeds the influx from the atmosphere (Visser and Voesenek, 2005). Such lack of oxygen is rapidly reflected as a drop in the soil redox potential (Ponnamperuma, 1972). Plants react to soil oxygen deprivation through a series of anatomical, morphological and physiological responses to mitigate the effects of soil anoxia. Soon after waterlogging, root metabolism shifts from aerobic respiration to the less efficient fermentation to produce energy, leading to a reduction in ATP production (Gibbs and Greenway, 2003). The energy deficit at root level results in a lack of phosphorylation of aquaporins, a reaction necessary to allow these proteins to regulate cell water flux, so root hydraulic conductivity is severely reduced (Tan et al., 2018). Some species have the capacity to grow adventitious roots with aerenchyma, facilitating the diffusion of oxygen from the shoot into and along the roots, which allows cells to continue respiration and water and nutrient uptake (Armstrong, 1979; Justin and Armstrong, 1987; Colmer, 2003; Striker et al., 2007; Colmer and Greenway, 2011). The species studied present variable ability to generate adventitious roots with aerenchyma and thus a high gas-filled volume (porosity); root porosity in wheat can reach values of 19–30% (Huang et al., 1994) and in barley 7–23% (Pang et al., 2004), whereas roots of rapeseed (Voesenek et al., 1999) and of field pea (Healy and Armstrong, 1972) did not develop aerenchyma when in an oxygen deficient medium.

The impaired root functioning under waterlogging impacts the physiological responses of the shoots, particularly the carbon fixation. Waterlogging-induced partial stomatal closure could constrain internal CO_2_ levels and limit carbon fixation (Malik et al., 2001; Striker et al., 2005), but this variable has rarely been quantified (e.g., in wheat, Wu et al., 2014; in rapeseed, Leul and Zhou, 1998). Apart from stomatal limitations, photosynthesis rates can also be determined by non-stomatal factors, such as mesophyll conductance (Jones, 1985; Flexas and Medrano, 2002), damage to photosystem II (PSII) caused by reactive oxygen species (ROS) (Nishiyama et al., 2006; Ashraf, 2012), and leaf chlorosis related to chlorophyll degradation due to an accelerated leaf senescence (Hörtensteiner, 2006; Araki et al., 2012). Although there are reports regarding the impact of waterlogging on carbon fixation of the winter crops in this study (e.g., Huang et al., 1994 for wheat; Pang et al., 2004 for barley; Leul and Zhou, 1998 for rapeseed, and Malik et al., 2015 for field pea), comparisons between these species are scarce (see de San Celedonio et al., 2017 for wheat and barley).

Low photosynthesis rates can be a constraint to shoot and root growth, and ultimately reduce seed production (Sinclair and Horie, 1989). Only some studies have analyzed the impact of waterlogging throughout the entire plant life-cycle, describing responses in vegetative growth and seed production. As examples: in wheat, 20 days of waterlogging on 3–4 leaf-stage plants resulted in a final dry mass and yield representing 95 and 90% of controls, respectively (Collaku and Harrison, 2002; Pampana et al., 2016a); in barley, plants attained 85 and 90% of controls in dry mass and yield when waterlogged for 20 days at 3–4 leaf-stage (Masoni et al., 2016); in rapeseed, 21 days of soil hypoxia, applied to 5-leaf stage plants, constrained growth as the stressed individuals attained 77% in dry mass and 73% in yield compared to the control (Leul and Zhou, 1998); in field pea, 5 days of waterlogging had a substantial impact on plant dry mass accumulation and seed production as, respectively, stressed plants attained 35–50% and 5–25% of controls (Jackson, 1979; Pampana et al., 2016b). Nevertheless, information on root and shoot growth rates during waterlogging and subsequent recovery is scarce, and studies to functionally link leaf physiological responses with growth (i.e., RGR) and seed production (but see Li et al., 2011 for wheat are few). In addition, waterlogging can affect plants differently depending on the growth stage (e.g., de San Celedonio et al., 2014 for wheat and barley; Gutierrez Boem et al., 1996 for rapeseed; Belford et al., 1980 for field pea). de San Celedonio et al. (2014) found that seed production is more compromised in wheat and barley when waterlogged at late stages (e.g., around flowering) as compared with earlier stages, which probably relates to the importance of photoassimilate supply during these stages. A rise in temperatures and air vapor pressure deficit (VPD_air_) during the later stages (i.e., mid-late spring) as compared to earlier ones (i.e., late winter to early spring), could also exacerbate the effects of waterlogging.

This study evaluated the tolerances to waterlogging in four widely used winter crops: wheat, barley, rapeseed and field pea. Plants of these species were exposed to a 14-day waterlogging period imposed at an ‘early’ or a ‘late’ plant stage. In addition, to shed light on the factors limiting growth, a suite of parameters that influence carbon fixation, such as stomatal and mesophyll conductances, leaf greenness and damage to PSII, along with internal CO_2_ were examined. These parameters, as well as root aerenchyma and growth, were measured not only during the waterlogging periods but also after, so that plant recovery was also assessed to maturity, as well as the resulting seed production.

## Materials and Methods

### Site and Plant Material

The experiment was carried out at the School of Agriculture, University of Buenos Aires, Argentina (34° 35′ S, 58° 29′ W), under outdoor conditions during July to November of 2016 [VPD_air_, maximum (*T*_max_) and minimum (*T*_min_) air temperature and photoperiod in Supplementary Figure S1]. Seeds of wheat (*Triticum aestivum*, cv. AGP FAST from Buck breeder, Argentina), barley (*Hordeum vulgare*, cv. Andreia from Quilmes malting, Argentina), rapeseed (*Brassica napus*, cv. Hyola 575 CL, from Advanta Seeds, Argentina) and field pea (*Pisum sativum*, cv. Viper from AFA-Federated Argentinean Farmers Society, Argentina) were sown on July 12th. The varieties chosen, widely grown in Argentina, have similar durations of life cycle (i.e., flowering and maturity).

### Experimental Design

Plants were cultivated in polyvinyl chloride (PVC) tubes of 66 cm length and 10 cm diameter, with a capacity of 5.2 L. The bottom end of each PVC tube was fitted with a fine mesh which retained the soil but allowed water drainage (or entry during waterlogging, see below). Tubes were placed in 1 m side plastic cubical containers, which had a valve located at the bottom (so waterlogging and drainage could be regulated). Each tube was filled with a mixture (3:1) of sand and silty clay loam soil (Typic Argiudoll). 60 tubes were used per species (240 tubes in total). Three seeds per tube were sown, and after 1 week, seedlings were thinned to one per tube. Before sowing, field pea seeds were inoculated with *Rhizobium leguminosarum* biovar *viceae* (Signum, Rizobacter^®^) and sprayed with fungicide [Maxim XL, Syngenta^®^ (Mefenoxam+fludioxonil)] as a common agronomic practice. Fertilizer applications are described below. Tubes were kept free of weeds by hand removal and any diseases and insect pests were controlled as required, by applying fungicides [Reflect Xtra, Syngenta^®^ (isopyrazam+azoxystrobin) for wheat and barley; Orquesta, BASF^®^ (fluxapyroxad+epoxiconazole+pyraclostrobin) for field pea, and K Mamboretá^®^ (captan) for rapeseed, at 50 and 80 days after sowing (DAS)] and insecticides [D Mamboretá^®^ (dimethoate) for wheat, barley and rapeseed at 55 and 104 DAS].

Three treatments were applied to plants: (i) well-drained controls watered daily and allowed to drain freely, (ii) waterlogged at an ‘early’ stage and (iii) waterlogged at a ‘late’ stage. Early-waterlogging, which coincided with the vegetative stage, was imposed at 65 DAS. Containers with their valves closed were filled with tap water for 14 days to reach 1–2 cm of water above the soil level of the tubes. After the waterlogging period ended, valves were opened so that the water drained and from then onwards, plants were watered daily to field capacity until the end of the experiment, to assess their recovery. Late-waterlogging, which occurred during plant reproductive stages (85 DAS for wheat, barley and rapeseed; 87 DAS for field pea), also lasted 14 days, and again recovery post-waterlogging was also monitored. In both waterlogging treatments, the purpose of having all species waterlogged at the same time was to have reliable comparisons among them, as differences in the environmental conditions [e.g., temperature and atmospheric vapor pressure deficit (VPD_air_)] if waterlogged at different moments could differentially impact on plant responses to waterlogging (e.g., Grassini et al., 2007; de San Celedonio et al., 2014).

Fertilizer (Nitrofull Emerger^®^, Argentina: 12% N, 5% P, 15% K, 2% Mg, 8% S, 3% Ca, 0.02% Zn, 0.2% Fe, 0.02% Mn and 0.015% Bo; % are by weight) was applied to the substrate of all tubes, distributed in three doses of 0.7 g each (2.1 g total per tube). This total amount per tube was based on providing N at a level equivalent to 200 kg per ha (typical dose used under field conditions). In all treatments, the first dose was added 58 DAS (1 week before early-waterlogging), the second dose was applied at 80 DAS (to coincide with 1 day after early waterlogging period ended), and the third dose was applied 102 DAS (to coincide with the start of recovery period after the late waterlogging). The split-application was aimed at reducing potential effects of any nutrient leaching from the tubes either by watering or by waterlogging and subsequent drainage.

### Measurements

#### Environmental Growing Conditions

Air temperature and relative humidity were monitored with an automatic meteorological station (Davis Vantage Pro2, CA, United States) at the site of the experiment. VPD_air_ was estimated as the difference between the actual air vapor pressure (*e*_a_) and the saturated vapor pressure (*e*_s_) using the Clausius–Clapeyron equation. VPD_air_ values ranged between 0.49 and 0.89 kPa during early-waterlogging and between 0.69 and 1.07 kPa during late-waterlogging. Air mean temperatures ranged between 10.4 and 20.2°C during early-waterlogging and between 15 and 21.6°C during late-waterlogging (Supplementary Figure S1).

The redox potential of the substrate in control and waterlogged conditions was measured with a redox electrode (Fiedler et al., 2007). The soil pH, measured at both conditions, was used to transform the readings to Eh at pH 7 (Eh_7_) assuming a slope of 59 mV per pH unit (Bohn, 1971). Values for Eh_7_ dropped from 400–440 mV in drained conditions to 110–124 mV after 1 week of waterlogging and remained between 109 and 121 mV by the end of the 2nd week of waterlogging. Five days after waterlogging ceased was enough to allow Eh_7_ to recover to similar values to those of the well-drained control tubes. The evolution of Eh_7_ (reduction and recovery) was similar for both early- and late-waterlogging events (Supplementary Figure S2).

The flowering period of each species was described by dating the beginning and end of flowering. The approximate beginning of seed filling was also recorded. These stages were described according to the status of at least 50% of the plants of each species (Supplementary Figure S3).

#### Leaf Physiological Measurements

Net photosynthesis (*P*_n_), stomatal conductance (*g*_s_) and internal CO_2_ (*C*_i_) were measured on the topmost fully expanded leaves of control and waterlogged plants, from the beginning of the treatments until the end of the experiment. Measurements were taken using a portable infrared gas analyser ((IRGA) Li-Cor 6400, Li-Cor Inc., Lincoln, NE, United States) under saturating light of 1500 μmol m^-2^ s^-1^ PPFD provided by the 6400-40 leaf chamber fluorometer using a mix of 90% red and 10% blue light. Air flow (300 mmol s^-1^) and CO_2_ concentration (400 μmol mol^-1^) in the reference chamber and block temperature (24°C) were automatically controlled. Mesophyll conductance (*g*_m_) was calculated using the equation from Bernacchi et al., 2002:

$$g_{m}=\frac{P_{n}}{C_{i}=\frac{\Gamma *\left(J+8\left(P_{n}+R_{d}\right)\right)}{J-4\left(P_{n}-R_{d}\right)}}$$

where *P*_n_, *C*_i_, and *J* are net photosynthesis, leaf internal CO_2_ concentration and electron transport rate, respectively. Leaf *R_d_* (day respiration) values were extracted from the literature (Barbour et al., 2010; Shrestha, 2017; Walker et al., 2017). Γ^∗^ is the specificity factor of Rubisco for CO_2_ and O_2_ and was estimated from the response of Γ^∗^ to temperature as described by Bernacchi et al. (2002) by using the leaf temperature measured by the thermocouple in the chamber of the Li-Cor 6400.

Leaf greenness was measured in young (top-most fully expanded leaf) and adult-basal leaves (lower third of the plant) using a chlorophyll meter (SPAD-502, Konica Minolta Sensing Inc., Osaka, Japan). This parameter is useful to examine the effects of waterlogging on leaf yellowing, associated with N remobilisation and senescence in relation to leaf age among the tested species.

Maximum quantum efficiency of PSII (*F*_v_/*F*_m_) was measured on top-most fully expanded leaves after a dark-adaptation period of 30 min by using leaf-clips and the OS-30p portable fluorometer (Opti-Sciences Inc., United States). This parameter indicates the proportion of functional PSII reaction centers, so that it can be used to quantify the degree of photoinhibition (Maxwell and Johnson, 2000).

All IRGA measurements were taken two times per week during waterlogging, and once a week during the recovery period, and four replicates were used. SPAD and *F*_v_/*F*_m_ measurements were taken two times per week for the entire duration of the experiment, and five replicates were used for each of these measurements.

#### Root Aerenchyma Formation

Aerenchyma formation was assessed in adventitious roots (white colored, 3.5–4 cm minimum length) of all species taken from control and waterlogged plants at the end of early- and late-waterlogging (*n* = 4 per species and treatment combination). Root segments of 2.5–3 cm were preserved in 70% ethanol, and then segments of 1 cm length were taken 2 cm from the apex and dehydrated in a series of increasing ethanol concentrations and then embedded in paraffin wax. Cross sections of 7–8 μm thick were cut using a steel blade and rotary microtome (Leica RM 2235, Leica Microsystems, Germany), then stained with safranin and fast green and mounted on Canada balsam. Representative images of root sections were photographed using an optical microscope (Zeiss Axioplan; Zeiss, Oberkochen, Germany) connected to a digital camera. The percentage occupied by aerenchyma in each cross-section was determined by adding up the areas of all present lacunae, to then divide it by the total area of the cross-section (Striker et al., 2014). These areas were quantified by hand using the free software ImageJ version 1.47 (National Institutes of Health: Bethesda, MD, United States).

#### Plant Growth, Dry Mass and Seed Production Responses

Plants were harvested: (i) at the beginning of early-waterlogging (65 DAS), (ii) at the end of early-waterlogging (79 DAS), (iii) when late-waterlogging started (85 DAS with the exception of field pea, which was harvested at 87 DAS), (iv) at the end of late-waterlogging (99 DAS with exception of field pea, which was harvested at 101 DAS) and (v) at maturity. Six replicates were sampled for each species and treatments at all harvests. Each plant was divided into roots, shoots and, in case of mature plants, also seeds. All harvested material was oven dried at 57°C for 72 h and weighed. The relative growth rates (RGR) were calculated for shoots and roots following the procedure by Hunt (1982).

### Statistical Analyses

Physiological variables (*A*, *g*_s_, *g*_m_, *C*_i_, SPAD_adult leaf_, SPAD_young leaf_, and *F*_v_/*F*_m_) were analyzed by a three-way ANOVA with ‘species,’ ‘treatment’ and ‘time’ as main factors. Calculated RGR values were compared between controls and each waterlogging treatment for each species and period of analysis using Student’s *t*-test (degrees of freedom = 10).

Root aerenchyma percentages and dry mass responses were analyzed by a two-way ANOVA with ‘species’ and ‘treatment’ as main factors. Additional contrasts using Fisher’s LSD test were performed to compare treatments within species for shoot, root, and seed dry masses. Normality and homogeneity of variances of the data were checked before ANOVAs. Statistical analyses were performed using Infostat software (Di Rienzo et al., 2011) and graphs were made with GraphPad Prism 5 for Windows PRISM, GraphPad. 5 (2009)^1^.

## Results

### Waterlogging Affected Species Differentially for Leaf Physiology and Growth During Early and Late Life-Cycle Stages

**FIGURE 1:**
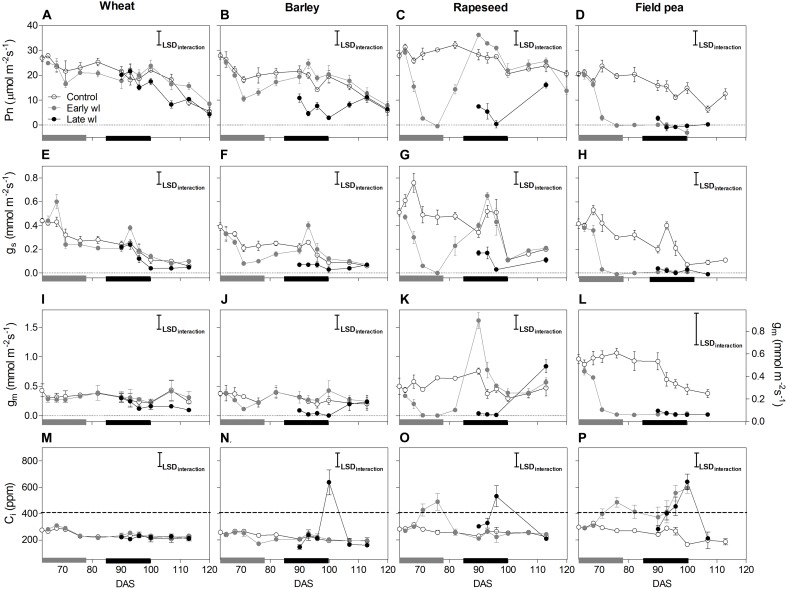
Net photosynthesis (*P*_n_: **A–D**), stomatal conductance (*g*_s_: **E–H**), mesophyll conductance (*g*_m_: **I–L**) and internal CO_2_ (*C*_i_: **M–P**) of control, early-waterlogged (Early wl) and late-waterlogged (Late wl) plants of wheat **(A,E,I,M)**, barley **(B,F,J,N)**, rapeseed **(C,G,K,O)** and field pea **(D,H,L,P)** on the top-most fully expanded leaves, over time (DAS is days after sowing). Note that the scale for *g*_m_ of field pea **(L)** differs to those of the other species **(I–K)**. Measurements were taken under saturating light of 1500 mmol m^-2^ s^-1^ PPFD provided by the 6400-40 leaf chamber fluorometer using a mix of 90% red and 10% blue light. Air flow, CO_2_ concentration in the reference chamber and block temperature were automatically controlled by the equipment at 300 mmol s^-1^, 400 μmol mol^-1^ (ppm) and 24°C, respectively. Gray and black bars on the *x*-axis represent the 14-day early- and late- waterlogging periods, respectively. The dashed horizontal line in **(M–P)** indicates external CO_2_. Pn LSD_interaction_ = 4.99 μmol m^-2^ s^-1^; *g*_s_ LSD_interaction_ = 0.10 mmol m^-2^ s^-1^; *g*_m_ LSD_interaction_ = 0.26 mmol m^-2^ s^-1^, *C*_i_ LSD_interaction_ = 111 ppm. The bars represent the LSD (Fisher’s protected least significant difference at *P* = 0.05). ANOVA results are presented in Table 2. Values are means ± standard errors of four replicates. Measurements for rapeseed at 100 and 107 DAS are missing because of complete leaf abscission, after which the newly sprouted leaves were big enough to measure.

**FIGURE 2:**
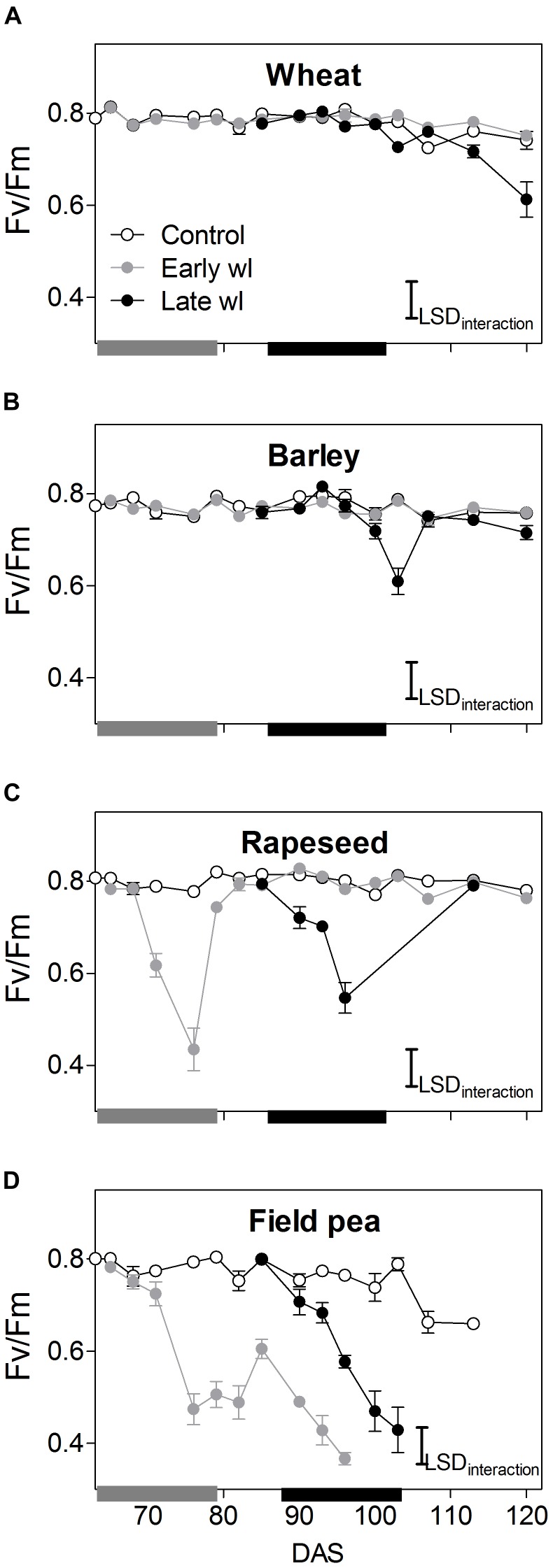
Chlorophyll fluorescence (*F*_v_/*F*_m_) of control, early-waterlogged (Early wl) and late- waterlogged (Late wl) plants of wheat **(A)**, barley **(B)**, rapeseed **(C)**, and field pea **(D)** on the top-most fully expanded leaves, over time (DAS is days after sowing). Gray and black bars on the *x*-axis represent the 14-day early- and late-waterlogging periods, respectively. *F*_v_/*F*_m_ LSD_interaction_ = 0.06. The bars represent the LSD (Fisher’s protected least significant difference at *P* = 0.05). ANOVA results are presented in Table 2. Values are means ± standard errors of five replicates. Measurements for rapeseed at 100, 103, and 107 DAS are missing because of complete leaf abscission, after which the newly sprouted leaves were big enough to measure.

**FIGURE 3:**
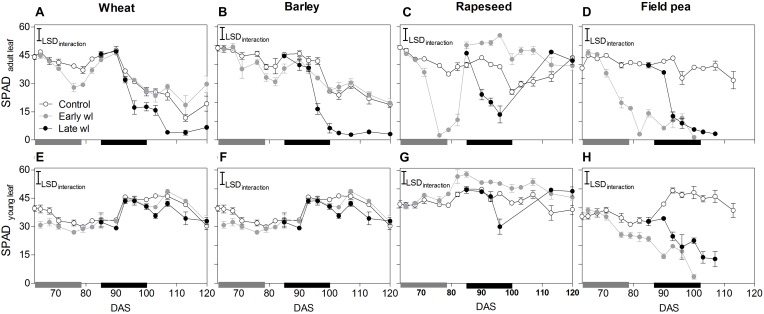
Leaf greenness of adult leaves located on the basal third of the plant (SPAD_adultleaf_: **A–D**) and from the top-most fully expanded leaf (SPAD_youngleaf_: **E–H**) of control, early- waterlogged (Early wl) and late-waterlogged (Late wl) plants of wheat **(A,E)**, barley **(B,F)**, rapeseed **(C,G)** and field pea **(D,H)**, over time (DAS is days after sowing). Gray and black bars on the *x*-axis represent the 14-day early- and late-waterlogging periods, respectively. SPAD_adultleaf_ LSD_interaction_ = 5.90, SPAD_youngleaf_ LSD_interaction_ = 5.79. The bars represent the LSD (Fisher’s protected least significant difference at *P* = 0.05). Values are means ± standard errors of five replicates. Measurements for rapeseed at 100, 103, and 107 DAS are missing because of complete leaf abscission, after which the newly sprouted leaves were big enough to measure.

**FIGURE 4:**
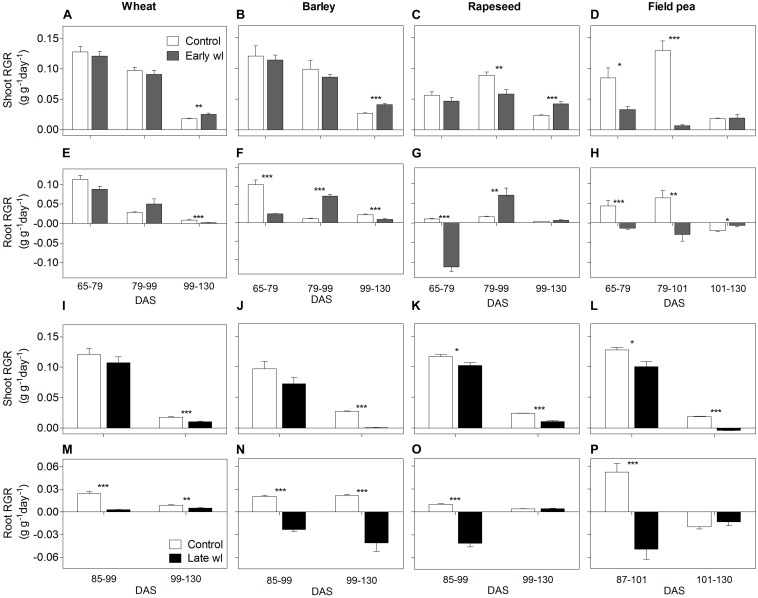
Shoot and root relative growth rate (RGR) of control vs. early-waterlogged plants (Early wl) (**A–D**; **E–H**, respectively) and control vs. late-waterlogged plants (Late wl) (**I–L**; **M–P**, respectively) of wheat **(A,E,I,M)**, barley **(B,F,J,N)**, rapeseed **(C,G,K,O)**, and field pea **(D,H,L,P)**, over time (DAS is days after sowing). RGRs comparisons from control vs. early-waterlogged plants considered three periods: early-waterlogging [65–79 days after sowing (DAS)], early recovery (79–99 DAS excepting for field pea, which occurred at 79–101 DAS) and late recovery (99–130 DAS excepting for field pea, which occurred 101–130 DAS). RGRs comparisons from controls vs. late-waterlogged plants considered two periods: late-waterlogging (85–99 DAS excepting for field pea, which was during 87–101 DAS) and the recovery until the end of the experiment (99–130 DAS excepting for field pea, which lasted from 101 to 130 DAS). Asterisks indicate significant differences between treatments within species (^∗^*P* = 0.05, ^∗∗^*P* = 0.01, ^∗∗∗^*P* = 0.001). Values are means ± standard errors of 6 replicates.

Waterlogging affected differently all leaf physiological parameters measured and, thereby shoot and root growth along the experiment, depending on the species and the timing of the stress (Figures 1–4, significant ‘species × treatment’, ‘treatment × time’ and ‘species × treatment × time’ interactions in Table 1).

**Table 1:** *F*-values of three-way ANOVA (factors: ‘species’, ‘treatment’, and ‘time’) for net photosynthesis (*P*_n_), stomatal conductance (*g*_s_), mesophyll conductance (*g*_m_), internal CO_2_ (*C*_i_), *F*_v_/*F*_m_, and SPAD values of adult and young leaves of control, early- and late- waterlogged plants of wheat, barley, rapeseed and field pea^A^.

Variables	Species (Sp)	Treatment (T)	Time	Sp × T	Sp × time	T × time	Sp × T × time
Net photosynthesis	255.30***	287.27***	51.06***	57.23***	6.41***	17.24***	5.43***
Stomatal conductance	98.47***	132.55***	110.23***	31.85***	3.93***	13.30***	4.43***
Mesophyll conductance	65.04***	31.62***	3.83***	9.88***	2.25***	3.42***	3.17***
Internal CO_2_	55.51***	25.90***	23.01***	9.88***	3.06***	6.05***	2.73***
*F*_v_/*F*_m_	196.81***	134.90***	15.88***	75.36***	8.98***	7.62***	6.93***
SPAD_adultleaf_	92.40***	480.42***	152.85***	68.18***	25.77***	21.56***	13.08***
SPAD_youngleaf_	269.69***	120.42***	21.64***	58.92***	10.77***	7.77***	5.99***


*^∗∗∗^P = 0.001. ^*A*^Degrees of freedom for each source of variation were: 3 (‘species’), 2 (‘treatment’), 6 (‘species × treatment’). 12, 35, 18, 46, and 360 (‘time,’ ‘species × time,’ ‘treatment’ × ‘time,’ ‘species × treatment × time’ and ‘error’ for P_*n*_, g_*s*_, g_*m*_, and C_*i*_, respectively). 15, 44, 23, 61, and 536 (‘time,’ ‘species × time,’ ‘treatment’ × ‘time,’ ‘species × treatment × time’ and ‘error’ for F_*v*_/F_*m*_, SPAD of adult and young leaves, respectively).*

In wheat, the early-waterlogging did not affect physiological performance. Values measured for stomatal conductance (*g*_s_), mesophyll conductance (*g*_m_), internal CO_2_ (*C*_i_) (Figures 1E,I,M) and *F*_v_/*F*_m_ (Figure 2A) showed similar patterns with time for both the early-waterlogged and control plants. In agreement, there were no differences between treatments in net photosynthesis (*P*_n_) and relative growth rates (RGRs) for shoot and roots (Figures 1A, 4A,E). Only slightly lower leaf greenness values (SPAD) for adult leaves (85% of controls) were evident in waterlogged plants by the end of the stress (Figures 3A,E). Through the recovery period, plants were able to restore SPAD values 5–7 days after the waterlogging was removed (Figures 3A,E), coinciding with the maintenance of the rest of the parameters until maturity (Figures 1A,E,I,M, 2A). Interestingly, 73% higher *P*_n_ values compared to controls were observed in previously waterlogged plants 1 week before maturity (Figure 1A), which also showed a 42% higher shoot RGR compared to controls during the 99–130 DAS period (Figure 4A).

Similarly to what occurred in early-waterlogging, during late-waterlogging of wheat no differences in any physiological variable were observed, as *g*_s_, *g*_m_, *C*_i_, *F*_v_/*F*_m_, SPAD, and *P*_n_ remained similar to controls (Figures 1A,E,M, 2A, 3A,E). Along recovery, physiological parameters maintained similar to controls, except for SPAD in adult leaves which began to decline until near maturity, reaching 18% of controls 1 week after water subsided; denoting accelerated plant senescence (Figure 3A). Although root growth was 13% of controls during late-waterlogging, shoot RGR presented no differences compared to controls; and during recovery root and shoot RGR attained 63 and 59% of controls, respectively (Figures 4I,M).

In barley, early-waterlogging induced changes in leaf physiology, such as *g*_s_ reduced to 38% of controls from 1 week of waterlogging (Figure 1F), followed by *C*_i_ lessened to 73% of controls by the end of the stress (Figure 1N); however, there were no changes either in *g*_m_ or *F*_v_/*F*_m_. *P*_n_ was reduced to 58% of controls 1 week after waterlogging was imposed, likely due to stomatal limitations (Figure 1B). Waterlogged plants showed a trend toward low SPAD values in adult and young leaves during the stress (Figures 3B,F). Shoot growth was not affected but root RGR was reduced to 33% of controls due to early-waterlogging. Afterwards, during the recovery, barley showed an important ability to restore its *g*_s_, *C*_i_, and SPAD values, along with a full recovery of *P*_n_ (similar to controls) in only 1 week after removing the stress (Figures 1B,F,N, 3B,F). In line with this, a 5.7-fold higher root RGR during the 79–99 DAS recovery period was observed concerning controls, and subsequently, shoot RGR was 53% higher than controls during the 99–130 DAS period (Figures 4B,F).

Late-waterlogging resulted in more adverse effects on the physiology and growth of barley, compared to early-waterlogging. Firstly, a decrease in *g*_s_ (values attained 27% of controls) occurred 5 days after waterlogging (Figure 1F), and then *g*_m_ had a drastic reduction 1 week after waterlogging started, attaining 9% of controls (Figure 1J). In addition, *F*_v_/*F*_m_ dropped by 77% of controls at the end of the stress, indicating damage to PSII (Figure 2B), which concurred with a 5.7-fold raise in *C*_i_ by the end of waterlogging (Figure 1N). In line with these responses, plants showed reductions in *P*_n_ 5 days after waterlogging was imposed (55–15% of controls) (Figure 1B). A progressive fall in SPAD of adult leaves occurred 1 week after waterlogging until reaching minimum values (3.7–6.3 SPAD units) at the end of the stress (Figure 3B). Such poor physiological performance during the stress was in turn related to a negative root RGR due to root mortality (Figure 4N). During the recovery, *g*_s_ and *g*_m_ of previously waterlogged plants could not reach control values (Figures 1F,J), although *C*_i_ was fully restored a few days after the stress (Figure 1N). Adult leaves continued with very low SPAD values (i.e., senescing leaves) (Figure 3B). Despite *F*_v_/*F*_m_ was restored during recovery (Figure 2B), *P*_n_ values could not be re-established up to control levels until 1 week before maturity (Figure 1B). So, plants were not able to resume root growth (RGR was still negative) and shoot RGR was close to zero, both symptoms of an approaching end of the plant life-cycle (Figures 4J,N).

Rapeseed showed important changes in leaf physiology due to early-waterlogging, as *g*_s_ was drastically reduced during the stress period, reaching values close to zero from 1 week after waterlogging (Figure 1G). A reduction in *g*_m_ occurred 3 days after waterlogging, to continue dropping until reaching minimum values 1 week after the beginning of the stress (Figure 1K). Simultaneously, early-waterlogged plants presented *F*_v_/*F*_m_ values 63% of controls, indicating PSII damage (Figure 2C), followed by a rise of 2.1-fold higher than controls in *C*_i_ (Figure 1O). At the same time, *P*_n_ was progressively reduced during waterlogging until reaching values close to zero. Early-waterlogged plants showed a drastic reduction in SPAD values on adult leaves, indicating early leaf senescence (Figure 3C) and most of the leaves dropped off by the end of the stress. Waterlogging had a drastic impact on root growth also, as root RGR was negative, caused mainly by tissue death during waterlogging (Figure 4G). However, during recovery, rapeseed showed great ability to sprout buds (i.e., branching) soon after the stress was removed, allowing re-establishing growth from new leaves. Two weeks after waterlogging removal, *g*_s_ and *g*_m_ of these new leaves exhibited a peak, reaching higher values than controls (125 and 212% of controls, respectively), to later remain similar to controls until maturity (Figures 1G,K). *F*_v_/*F*_m_ and *C*_i_ of these new leaves showed similar values than controls since waterlogging was removed (Figure 2C), while SPAD values were 43% higher than those of controls, a pattern that persisted until maturity (Figure 3C). This recovery allowed reaching *P*_n_ values 28% higher than controls 2 weeks after waterlogging, remaining similar to controls onwards (Figure 1C). During the 79–99 DAS recovery period, root RGR was 3.6-fold higher compared to controls (Figure 4C) while shoot RGR was 65% of controls. Later, during 99–130 DAS, shoot RGR in recovered plants was 79% higher than the controls (Figure 4G).

As in early-waterlogging, rapeseed plants exposed to waterlogging at late-stage showed important differences in leaf physiological performance and growth during the stress. Reductions in *g*_s_ and *g*_m_ occurred 5 days after the stress was imposed, reaching values close to zero by 1 week of waterlogging (Figures 1G,K). Additionally, significant differences for leaf greenness were observed, not only in adult leaves but also in younger ones (65 and 37% of controls, respectively) (Figures 3C,G). Anticipated leaf senescence was followed by abscission both in young and adult leaves (abscised leaves were included in the quantification of shoot dry mass). In addition, *F*_v_/*F*_m_ values stood around 65% of controls throughout late-waterlogging indicating damage to the PSII (Figure 2C), in coincidence with higher *C*_i_ values showing a peak at the end of waterlogging (2.2-fold higher) (Figure 1O). Drastic reductions in *P*_n_ were observed, showing values close to zero 1 week after waterlogging was imposed (Figure 1C). Such a poor performance during waterlogging was evidenced by a negative root RGR (Figure 4O). After water was removed, plants showed partial capacity to recover from the stress due to bud sprouting; *g*_s_ and *g*_m_ of new leaves attained 55% and similar values of controls, respectively (Figures 1G,K). Additionally, after 1 week of recovery, leaf greenness of the newly produced leaves reached higher values than controls (Figures 3C,G). Also, *F*_v_/*F*_m_ and *C*_i_ values were restored (Figures 1O, 2C) and *P*_n_ attained 67% of controls near maturity (Figure 1C). However, as the time to recover from the late-waterlogging (late-stage) was shorter than for the early-stage, the recovery was lower. Shoot RGR represented 56% of controls while root RGR was low and similar between treatments in line with the end of the plants life-cycle (Figures 4K,O).

In field pea, early-waterlogging severely affected leaf physiology and growth. Values close to zero were observed in *g*_s_ 1 week after waterlogging started, along with a similar pattern in *g*_m_ (Figures 1H,L). A progressive decline in SPAD of adult leaves was observed 1 week after waterlogging was applied, joined by decreases in SPAD of young leaves at the end of the stress (Figures 3D,H). *F*_v_/*F*_m_ declined to 49% of controls (i.e., damage to PSII), and a rise in *C*_i_ occurred at the same time (twofold higher compared to controls) (Figures 1P, 2D) matching the near to zero values for *P*_n_ (Figure 1D). Field pea had not only its root RGR affected, but also its shoot RGR reduced during early-waterlogging (*ca.* 38% of controls) (Figures 4D,H). During the recovery, field pea was not able to restore leaf functioning (Figures 1D,H,L,P, 2D, 3D,H). Root RGR continued showing negative values throughout the experiment, and shoot RGR was even more reduced with respect to controls during the recovery than along the waterlogging period (5% of controls in 79–101 DAS) (Figures 4D,H).

Field pea plants subjected to late-waterlogging behaved in a similar way to those exposed to this stress at the early-stage. Plants presented an abrupt fall in physiological performance soon after the stress was imposed, reaching values close to zero in *g*_s_ and *g*_m_ in only 3 days after the stress was imposed (Figures 1H,L). A drastic reduction in *F*_v_/*F*_m_ occurred 1 week after waterlogging (Figure 2D), as well as a rise in *C*_i_ (Figure 1P) and negligible values of *P*_n_ (Figure 1D). Additionally, SPAD values from both adult and young leaves were progressively reduced (Figures 3D,H) indicating early leaf senescence in waterlogged plants. Consequently, shoot RGR decreased during the stress (78% of controls) and plants presented negative root RGR (Figures 4L,P). Soon after waterlogging was removed, plants were not able to recover as revealed by the negative shoot and root RGR values (Figures 4L,P).

### Root Aerenchyma Formation Under Waterlogging Differed Among Species

**FIGURE 5:**
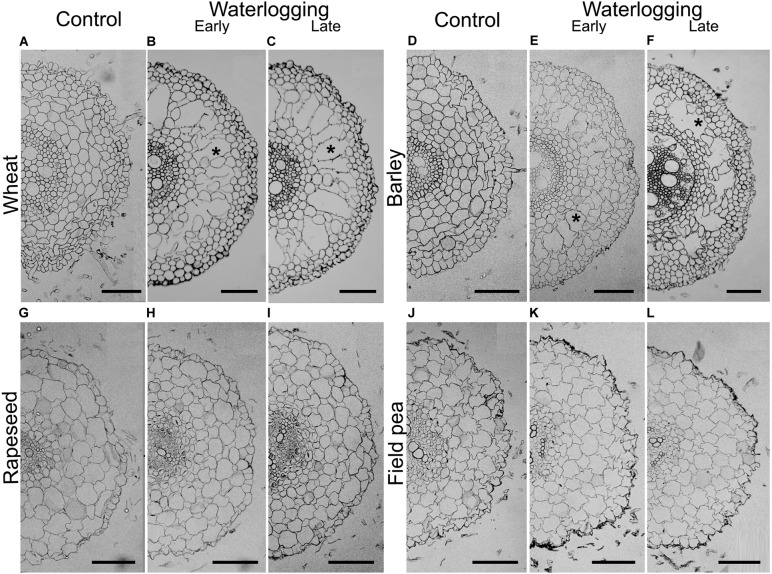
Adventitious root cross sections of control **(A,D,G,J)**, early **(B,E,H,K)** and late waterlogged **(C,F,I,L)** plants of wheat **(A–C)**, barley **(D–F)**, rapeseed **(G–I)** and field pea **(J–L)**. Cross-sections were taken at 20 mm from the root apex of 25–40-mm-roots. Asterisks denote examples of aerenchyma lacunae. Scale bars represent 100 μm. Values of aerenchyma percentage for wheat were 2.0 ± 0.6%, 20.4 ± 4.4%, and 22.2 ± 2.9% for control, early- and late-waterlogged plants, respectively. Barley presented values of 1.5 ± 0.5%, 18.9 ± 3.6%, and 13.9 ± 3.3% for control, early- and late-waterlogged plants, respectively. Rapeseed and field pea did not show any signs of aerenchyma formation under any treatment.

Aerenchyma formation in adventitious roots in response to waterlogging depended on the species (‘species × treatment’ interaction in Table 2). In wheat, waterlogging at early-stage induced the development of aerenchyma-lacunae in the root cortex, with an average of 20.4 ± 4.4% of the root cross-sectional area occupied by lacunae (Figure 5B). Wheat roots subjected to the stress at late stage showed a similar capacity to develop aerenchyma, with an average of 22.2 ± 2.9% (Figure 5C). Wheat roots from controls either from the early- or the late-waterlogging treatment contained low proportions of aerenchyma (2% on average) (Figure 5A).

**Table 2:** *F*-values of two-way ANOVA (factors: ‘species’ and ‘treatment’) for aerenchyma percentages in adventitious root cross sections as treatment responses of wheat, barley, rapeseed, and field pea.

	Early-waterlogging				Late-waterlogging		
			
	Species (Sp)	Treatment (T)	Sp × T		Species (Sp)	Treatment (T)	Sp × T
Total	22.67^∗∗∗^	59.18^∗∗∗^	20.24^∗∗∗^		25.14^∗∗∗^	68.09^∗∗∗^	25.14^∗∗∗^


*^*A*^^∗∗∗^: P = 0.001. Analyses were conducted separately on data at the end of each waterlogging period (control vs. early-waterlogged plants; control vs. late- waterlogged plants). ^*A*^Degrees of freedom for each source of variation were: 3 (‘species’), 1 (‘treatment’), 3 (Sp × T), and 24 (‘error’).*

In adventitious roots of barley, aerenchyma also formed in the cortex in response to waterlogging. Root samples from the early-waterlogging had lacunae occupying 18.9 ± 3.6% of the root cross-sectional area (Figure 5E). Late-waterlogging also triggered the formation of aerenchyma in the roots of barley, but the lacunae were 13.9 ± 3.3%, being slightly lower than for roots from the early-waterlogging treatment (Figure 5F) (Table 2). Contrastingly, roots of control plants for both, early- and late-waterlogging treatments, only presented 1.5% of aerenchyma on average (Figure 5D).

In rapeseed (Figures 5G–I) and field pea (Figures 5J–L), both species of relatively low waterlogging tolerance, in spite of forming relatively short adventitious roots (3–4 cm length) in the root-shoot junction, no noticeable root aerenchyma formation was found after neither early- nor late-waterlogging in the examined cross-sections. Roots from control plants evaluated at the same times when each waterlogging period ended also did not contain aerenchyma.

### Dry Mass and Seed Mass Responses Are Affected by Early- and Late- Waterlogging

In wheat, waterlogging at the early-stage did not impact on shoot or root dry mass, but seed per plant produced was 86% of controls (Table 3). In contrast, late-waterlogging significantly reduced both root and shoot dry mass as they attained 75% of controls, and there was a reduction in seed mass (71% of controls) (Table 3).

**Table 3:** Shoot, root and seed dry mass (g per plant) of mature plants of wheat, barley, rapeseed and field pea under control, and after early-waterlogging (Early wl) and late-waterlogging (Late wl) treatments followed by a recovery period.

	Control	Early wl	Late wl
**Wheat**			
Shoot	22.4 ± 0.4 a	23.3 ± 0.9 (104) a	16.9 ± 1.1 (75) b
Root	5.3 ± 0.3 a	4.8 ± 0.02 (90) a	3.5 ± 0.3 (66) b
Seed	8.9 ± 0.3 a	7.6 ± 0.5 (86) b	6.3 ± 0.4 (71) c
**Barley**			
Shoot	29.9 ± 1.4 a	28.9 ± 1.6 (97) a	10.5 ± 1.3 (35) b
Root	7.5 ± 0.6 a	5.1 ± 1.0 (69) b	0.5 ± 0.1 (7) c
Seed	10.6 ± 0.4 a	9.0 ± 0.5 (85) b	3.4 ± 0.4 (32) c
**Rapeseed**			
Shoot	19.3 ± 0.5 a	16.3 ± 0.7 (84) b	10.4 ± 1.6 (54) c
Root	5.0 ± 0.3 a	3.0 ± 0.4 (60) b	2.5 ± 0.3 (50) b
Seed	5.7 ± 0.2 a	4.5 ± 0.1 (79) b	1.5 ± 0.3 (26) c
**Field pea**			
Shoot	13.7 ± 2.1 a	2.0 ± 0.3 (15) b	4.2 ± 1.0 (31) b
Root	0.9 ± 0.1 a	0.1 ± 0.02 (10) b	0.3 ± 0.1 (29) b
Seed	7.5 ± 0.8 a	0.3 ± 0.1 (4) b	0.6 ± 0.2 (8) b


**Values attained by plants following waterlogging and recovery periods are given as the percentage of controls in brackets. Different letters across a row denote significant differences among treatments within a species based on Fisher’s LSD test (P = 0.05). Values are means ± standard errors of 6 replicates.**

In barley, early-waterlogged plants attained 69% of controls in root dry mass, but shoots were unaffected. Seed mass of stressed plants represented 85% of controls (Table 3). Conversely, late-waterlogging caused a drastic reduction in dry masses of both roots and shoots (stressed plants attained 7 and 35% of controls, respectively), and these plants produced seed mass about 32% of controls (Table 3).

In rapeseed, early-waterlogging compromised root dry mass (60% of controls) along with shoot dry mass (84% of controls). Seed yield showed values of 79% of controls (Table 3). Late-waterlogging had a greater impact on growth than early-, as root and shoot dry masses represented 48 and 54% of controls, respectively. Seed yield was reduced to 26% of controls (Table 3).

Field pea was the most adversely impacted species by waterlogging. Early-waterlogging provoked great losses of root and shoot mass (plants attained 10 and 15% of controls, respectively) (Table 3). Late-waterlogging reduced these components to 29 and 31% of controls for roots and shoots, respectively (Table 3). Seed production was considerably reduced by both waterlogging treatments, where early- and late-waterlogged plants had only 4.4 and 9.5% of seed mass compared to controls (Table 3).

## Discussion

This study provides a comprehensive evaluation of the progressive effects of 2 weeks of waterlogging at early- or late-stages on wheat, barley, rapeseed and field pea through an integrated analysis of root aerenchyma formation, leaf carbon fixation and its regulators (i.e., stomatal or non-stomatal limitations), combined with responses in root and shoot growth monitored during the stress and the recovery periods, along with seed production at maturity.

### Wheat and Barley Developed Substantial Aerenchyma in Adventitious Roots While Rapeseed and Field Pea Did Not

Wheat and barley produced adventitious roots with considerable aerenchyma. In wheat, aerenchyma occupied 20% of the root cross sections in early-waterlogged plants and 22% in late-waterlogged plants, being comparable with 19–30% aerenchyma in adventitious roots of wheat when waterlogged for 17 days commencing at the 3-leaf stage (Huang et al., 1994), and concurring with the range of values summarized by Herzog et al. (2016). In barley, root aerenchyma of waterlogged plants was slightly lower than in wheat (19 and 13%, for early- and late-waterlogging) but in line with those reported in previous works (e.g., Pang et al., 2004; de San Celedonio et al., 2017). The presence of substantial root aerenchyma is known to facilitate tissue aeration (Armstrong, 1979), thereby it could be related to a lessened impact of root-zone hypoxia on plants’ physiological performance Colmer and Greenway, 2011), as for wheat and barley in the present study.

Rapeseed and field pea did not form aerenchyma in adventitious roots under any growing condition. The inability of rapeseed to generate aerenchyma concurs with one earlier study on seedlings in which the short adventitious roots formed when grown in agar had very low gas-filled porosity (Voesenek et al., 1999). For field pea, Healy and Armstrong (1972) suggested that this species is not able (or has a minimal capacity) to form aerenchyma in roots, as we also observed. The lack of aerenchyma in the adventitious roots of both rapeseed and field pea would likely explain the greater impact of waterlogging on their physiological and growth performance as compared with barley and wheat.

### Waterlogging Impacts on Carbon Fixation Differentially Among Winter Crops Through Stomatal and Non-stomatal Limitations

Wheat presented no differences compared to controls in its leaf physiological parameters during early- and late-waterlogging. So, photosynthesis, stomatal and mesophyll conductances, as well as chlorophyll fluorescence were not affected by 14 days of waterlogging. These results concur with those reported by de San Celedonio et al. (2017) for 45-day-old plants subjected to 15 days of waterlogging where no differences in photosynthesis were found at the end of the stress. However, other authors have observed reductions in photosynthesis and stomatal conductance of 85 and 80%, respectively, in 21-day-old plants after 14 days of waterlogging, successfully reestablishing values to controls after a 14-day-recovery (Malik et al., 2001). Moreover, despite the similar physiological performances for early- and late-waterlogged plants of wheat and controls in the present study, a recent review by Herzog et al. (2016) indicated variability in the responses of wheat to waterlogging in their analysis of experiments that involved 23 different cultivars exposed to a range of waterlogging treatments from 7 to 34 days (data were from 15 separate studies), showing a wide range in photosynthesis reductions from 7 to 85% of controls.

Barley showed reductions in photosynthesis (58% of controls) during early-waterlogging attributed to stomatal limitations, as revealed by a lower *g*_s_ (43% of controls) along with falls in internal CO_2_ (*C*_i_; 72% of controls). Partial closing of stomata during waterlogging for barley was also found by Pang et al. (2004), where *g*_s_ followed a similar pattern to that of photosynthesis in 4-leaf stage plants waterlogged for 21 days. Nevertheless, no data on *C*_i_ of waterlogged plants for barley were available to match with *g*_s_, so this study demonstrates that stomata closing can indeed constrain the *C*_i_ available for photosynthesis during early-waterlogging in barley. In line with our results where no damage to PSII (i.e., low *F*_v_/*F*_m_) was detected during waterlogging, Zeng et al. (2013) reported only a slight decrease in *F*_v_/*F*_m_ (*ca*. 10%) in 1-leaf stage seedlings of barley waterlogged for 14 days. Regarding to recovery, a study by Pang et al. (2004) showed a successful recovery for 5 out of 6 genotypes analyzed 1 week after water subsided; which agrees with plants fully reestablishing *g*_s_ and *P*_n_ after a 3-day-recovery in our work. During late-waterlogging, photosynthesis of waterlogged barley was further reduced down to values representing only 25% of controls. This was firstly caused by partial stomatal closure (*g*_s_ 32% of controls) that resulted in a lower *C*_i_ (71% of controls). After 1 week of waterlogging, non-stomatal constraints to photosynthesis were also evident, such as reduced *g*_m_ along with damage to the PSII (drops in *F*_v_/*F*_m_), which derived in a rise in *C*_i_ (3.2-fold of controls) by the end of waterlogging. Damage to PSII in waterlogged barley plants resulting in decreased rates of *P*_n_ were also registered by Pang et al. (2007). Regarding recovery, physiological variables were fully restored 2 weeks after water had drained from the soil, near maturity, which suggests that barley is able to recover the leaf physiological functioning even at advanced plant stages.

In rapeseed, waterlogging had a substantial impact on leaf physiological parameters during the early-stage. *P*_n_ reached values close to zero after 1 week of waterlogging, mainly related to non-stomatal factors, such as highly diminished *g*_m_ and lower *F*_v_/*F*_m_ (56% of controls), which led to an increase in *C*_i_ values up to 1.9-fold of controls. Taken together, these results indicate that the reduction in carbon fixation was not related to the lack of internal CO_2_ as substrate but associated with a damaged photosynthetic apparatus. High sensitivity to waterlogging exhibited by rapeseed was also found in 5-leaf stage plants waterlogged for 3 weeks, showing lower *P*_n_ and increased *C*_i_ (Leul and Zhou, 1998). All physiological parameters were fully restored in the new developed leaves when assessed 10 days after water subsided. These new leaves were the result of the ability of plants to produce new branches, as already reported for plants of rapeseed (cv. Avatar) after being waterlogged for 14 days at stem elongation stage (Wollmer et al., 2018). Late-waterlogging had a greater impact over physiology, since plants not only showed *P*_n_ values close to zero but also exhibited generalized senescence as even young leaves had low greenness. Afterwards, although generation of new leaves through branching was observed with 1 week of recovery, these leaves were not able to reach control values for the measured physiological variables.

Field pea exhibited a leaf physiological performance severely affected by waterlogging at both growth stages, typical of waterlogging-sensitive species. One week after waterlogging, a fall in *g*_s_ was observed, and photosynthesis reached values close to zero, being the latter caused more likely by non-stomatal constraints characterized by a diminished *g*_m_ followed by an increase of *C*_i_. In addition, damage to PSII was evident, reflecting another constraint for carbon fixation *per se*; as well as clear symptoms of chlorophyll degradation due to lower SPAD values in both adult and young leaves, as compared to controls. Previous reports showed that *g*_s_ resulted in being a variable very sensitive to waterlogging for field pea, given the fact that 9-leaf-stage plants after 4 days of waterlogging showed values of 26 and 52% of controls for adult and young leaves, respectively (Jackson, 1979). Nevertheless, this study indicates that stomatal closure seems not to be the primary constraint for photosynthesis as *C*_i_ values increased in comparison to controls. The increase in *C*_i_ appears to be related to the damage to PSII and potentially in the activity of Rubisco as suggested by the low *F*_v_/*F*_m_ along with highly reduced *g*_m_. In line with this high sensitivity to waterlogging, for both early- and late-waterlogging, there was irreversible damage to leaf physiology as none of the variables were restored during recovery.

### The Effect of Waterlogging on Plant Growth and Seed Production Is Determined by Species and Growth Stage

Wheat showed similar performance to controls in shoot and root growth during early-waterlogging and its recovery. During late-waterlogging, only root RGR was affected. However, despite that growth inhibition when waterlogged, root RGR increased during recovery until reaching 63% of the controls, and indicating ability of wheat for root growth resumption after the stress. Similar to our results, Malik et al. (2001) found a substantial decline in root growth due to 14 days of waterlogging on 21-day-old wheat plants (root RGR 26% of controls), but also a subsequent root growth recovery after water drainage. Seed production per plant varied between waterlogging treatments (as in de San Celedonio et al., 2014), being 86 and 71% of controls for early- and late-waterlogged plants, respectively. Lesser yield losses in wheat (near 92% of controls) were reported in Li et al. (2011), probably because of a shorter waterlogging (6 days) divided in three periods of 2 days each, at 7- and 9-leaf stage, and also in heading (i.e., spikes appearance). Nevertheless, the tolerance to waterlogging regarding seed production depends on the combination of environmental conditions and considered genotypes, apart from growth stage and duration of waterlogging (Herzog et al., 2016). As examples, 14-day-waterlogged plants of wheat at 22 days after sowing (cv. Wyalkatchem) showed reductions in seed weight per plant to 32% of controls (Robertson et al., 2009); while 15 days of waterlogging during flowering (cv. Karasu-90) carried reductions to 62% of controls in seed production (Olgun et al., 2008).

In barley, shoot RGR was unaffected during and after early-waterlogging, and although root growth was negatively impacted by waterlogging, such plants showed 7-fold greater root RGR than controls during recovery. Enhancement in root growth after water subsided was probably supported initially by consumption of carbon reserves (as seen by de San Celedonio et al., 2017 for 20-day-waterlogged plants), and subsequently aided by current assimilation after leaf physiological parameters were restored during the 2nd week of recovery. Late-waterlogging severely affected growth as root RGR was negative (denoting tissue mortality) during and after waterlogging, and shoot RGR values were close to zero during recovery. Related to the latter, greenness of adult leaves was significantly lower in waterlogged plants, leading to a detrimental carbon fixation performance at the plant level, and contributing to (partially) explain such a poorer growth. Chlorosis of basal leaves of 1/2-week-old barley waterlogged for 14 days could be associated with nitrogen remobilisation (Pang et al., 2007; Zeng et al., 2013). The impact on seed production was higher in late- than in early-waterlogging, attaining values of 32 and 85% of controls, probably because early-waterlogging allowed for an effective recovery of physiological and growth parameters by the time of the critical period for yield determination. Contrasting timing of waterlogging during the life-cycle can be a determining factor for barley yield, as also reported that waterlogging during tillering (25–45 days after emergence) caused 25% reduction in final seed mass while in pre-flowering (65–85 days after emergence) led to a 75% reduction of seed mass (de San Celedonio et al., 2014).

Through the early-waterlogging period rapeseed showed positive shoot RGR but presented negative root RGR. Hypoxia-induced root mortality during waterlogging along with consumption of taproot reserves and low carbon fixation at plant level due to leaf senescence (i.e., adult leaves turned purple and then senescent, see also Gutierrez Boem et al., 1996; Wollmer et al., 2018) would constrain root growth during the stress period. Importantly, root RGR exhibited 3.6-fold greater values compared to controls during recovery in coincidence with the restoration of the leaf physiological activity after the rapid sprouting of new leaves during the recovery. Concerning late-waterlogging, plants showed negative root RGR (i.e., root mortality) along with shoot RGR of 88% of controls during waterlogging, concurring with findings for 53-day-old rapeseed waterlogged for 14 days (Gutierrez Boem et al., 1996). During recovery, root growth was almost zero and shoot RGR was only 44% of controls. As an expected consequence of the adverse effects of waterlogging on leaf physiology and growth, seed production was reduced but varied according to the growth stage at which waterlogging occurred; early-waterlogged plants attained 79% of the seed mass of controls, while the ones waterlogged at the late-stage showed values of 27% of controls. As in barley, leaf physiology and growth in early-waterlogged rapeseed might have been restored by the time yield definition occurred, so seed production was substantially less affected compared with late-waterlogging. Observations by Wollmer et al. (2018) also found that the sensitivity of rapeseed to 14-day-waterlogging varied according to plants’ growth stage when waterlogged; as the stress applied at stem elongation led to a seed mass 75% of controls, while waterlogging at floral bud appearance caused a seed production of 85% of controls.

Field pea waterlogged at early- and late-stages showed lower shoot RGR than controls, not only during the stress, but also after water subsided. During recovery, previously late-waterlogged plants presented negative shoot RGR, denoting leaf detachment. In relation with root growth, both waterlogging treatments resulted in root mortality (i.e., negative RGR) and values could not be restored, continuing with tissue death until maturity. These results contrast with those of Cannell et al. (1979) in which growth stage was important in determining the effects of short-term (5-day) waterlogging on pea; as plants waterlogged at 24-day-old (vegetative stage) attained 41 and 47% of controls in shoot and seed dry mass, respectively, while when the stress was imposed on 47-day-old plants (flowering stage), these plants attained 34 and 25% of controls in shoot and seed dry mass. In our study, 2 weeks of water excess had severe adverse effects at both growth stages and so did not allow us to discriminate responses according to the growth stage. Similarly, another report showed dramatic reductions of shoot RGR (only 17% of controls) after exposing 35-day old plants of field pea to 1-week waterlogging when assessed 10 days after water subsided (Solaiman et al., 2007). The sensitivity of field pea to waterlogging is further highlighted by the detrimental effect on plant seed production, with values of 4 and 8% of controls for early- and late-waterlogging. Even waterlogging for 5 days at the beginning of flowering decreased seed production in field pea to 38% of controls (Pampana et al., 2016b).

## Conclusion

There were substantial differences among the four winter crop species in tolerance to waterlogging. Wheat leaf physiology and shoot growth were not significantly affected by waterlogging in early- or late-stages of growth, but seed production was reduced to 86 and 71% of controls when plants experienced early- and late-waterlogging, respectively. In barley and rapeseed the growth stage when the stress occurred was critical for determining the effects on leaf physiological performance, dry mass responses and seed production (seed mass was on average 82 and 29% of controls in early- and late-waterlogging, respectively). For barley during early-waterlogging, photosynthesis was compromised by stomatal limitations and root growth was impacted; however, upon drainage plants recovered. During late-waterlogging of barley, photosynthesis was also initially reduced by stomatal limitations but then by non-stomatal (i.e., lower *F*_v_/*F*_m_ and *g*_m_) effects, and negative root RGR (indicating death of root tissue) was also observed; physiological parameters were restored only near maturity, leading to an unsuccessful growth recovery. In rapeseed, photosynthesis was compromised by non-stomatal limitations and root RGR was negative in both waterlogging treatments, and there were differences between growth stages in the ability (and available time) to recover from the stress, as plants could only show an acceptable recovery after water subsided in early-waterlogging. Field pea was severely affected either by early- or late-waterlogging, attaining on average 6% of controls for seed mass. During both waterlogging treatments, photosynthesis was decreased by non-stomatal limitations and root and shoot growths were both also compromised, and recovery was poor. Therefore, this study contributes to the understanding of the differential tolerances to early- and late-waterlogging of wheat, barley, rapeseed and field pea, by integrating leaf physiological variables related to carbon fixation, the ability (or not) to form aerenchyma in adventitious roots, along with shoot and root growth during the stress and importantly also recovery post-waterlogging, and ultimately, the impact on plant seed production.

## Author Contributions

All authors listed above made a substantial, direct, and intellectual contribution to this work. RP, DM, TC, and GS designed the experiments. EP discussed physiological measurements and trained RP to perform them. RP wrote the first draft of the manuscript with GS. DM, EP, and TC made significant comments and inputs to successive versions of the paper. All authors read and approved the text for publication.

## Conflict of Interest Statement

The authors declare that the research was conducted in the absence of any commercial or financial relationships that could be construed as a potential conflict of interest.

## Footnotes

1 www.graphpad.com
