# Corrigendum: Waterlogging of Winter Crops at Early and Late Stages: Impacts on Leaf Physiology, Growth and Yield

**DOI:** 10.3389/fpls.2019.01806

**Published:** 2020-02-04

**Authors:** Rocío Antonella Ploschuk, Daniel Julio Miralles, Timothy David Colmer, Edmundo Leonardo Ploschuk, Gustavo Gabriel Striker

**Affiliations:** ^1^IFEVA, Facultad de Agronomía, Universidad de Buenos Aires, CONICET, Buenos Aires, Argentina; ^2^Faculty of Science, School of Agriculture and Environment, The University of Western Australia, Crawley, WA, Australia; ^3^Facultad de Agronomía, Cátedra de Cultivos Industriales, Universidad de Buenos Aires, Buenos Aires, Argentina

**Keywords:** waterlogging, crops, aerenchyma, photosynthesis, yield

In the original article, there was a mistake in **Table 3** and legend as published. There were some unintentional errors in the values of the reported shoot dry mass. The corrected **Table 3** and legend appears below.

**Table 3:** Shoot, root and seed dry mass (g per plant) of mature plants of wheat, barley, rapeseed and field pea under control, and after early-waterlogging (Early wl) and late-waterlogging (Late wl) treatments followed by a recovery period.

	Control	Early wl	Late wl
**Wheat**			
*Shoot*	22.4 ± 0.4 a	23.3 ± 0.9 (104) a	16.9 ± 1.1 (75) b
*Root*	5.3 ± 0.3 a	4.8 ± 0.02 (90) a	3.5 ± 0.3 (66) b
*Seed*	8.9 ± 0.3 a	7.6 ±0.5 (86) b	6.3 ±0.4 (71) c
**Barley**			
*Shoot*	29.9 ± 1.4 a	28.9 ± 1.6 (97) a	10.5 ± 1.3 (35) b
*Root*	7.5 ± 0.6 a	5.1 ± 1.0 (69) b	0.5 ± 0.1 (7) c
*Seed*	10.6 ± 0.4 a	9.0 ± 0.5 (85) b	3.4 ± 0.4 (32) c
**Rapeseed**			
*Shoot*	19.3 ± 0.5 a	16.3 ± 0.7 (84)b	10.4 ± 1.6 (54) c
*Root*	5.0 ± 0.3 a	3.0 ± 0.4 (60) b	2.5 ± 0.3 (50) b
*Seed*	5.7 ± 0.2 a	4.5 ± 0.1 (79) b	1.5 ± 0.3 (26) c
**Field pea**			
*Shoot*	13.7 ± 2.1 a	2.0 ± 0.3 (15) b	4.2 ± 1.0 (31) b
*Root*	0.9 ± 0.1 a	0.1 ± 0.02 (10) b	0.3 ± 0.1 (29) b
*Seed*	7.5 ± 0.8 a	0.3 ± 0.1 (4) b	0.6 ± 0.2 (8) b

Values attained by plants following waterlogging and recovery periods are given as the percentage of controls in brackets. Different letters across a row denote significant differences among treatments within a species based on Fisher’s LSD test (P = 0.05). Values are means ± standard errors of 6 replicates.

Further, due to the error reported above, a correction has also been made to the Results section, subsection Dry Mass and Seed Mass Responses Are Affected by Early- and Late Waterlogging, paragraphs one, two, and four:

“In wheat, waterlogging at the early-stage did not impact on shoot or root dry mass, but seed per plant produced was 86% of controls (**Table 3**). In contrast, late-waterlogging significantly reduced both root and shoot dry mass as they attained 75% of controls, and there was a reduction in seed mass (71% of controls) (**Table 3**).”

“In barley, early-waterlogged plants attained 69% of controls in root dry mass, but shoots were unaffected. Seed mass of stressed plants represented 85% of controls (**Table 3**). Conversely, late-waterlogging caused a drastic reduction in dry masses of both roots and shoots (stressed plants attained 7 and 35% of controls, respectively), and these plants produced seed mass about 32% of controls (**Table 3**).”

“Field pea was the most adversely impacted species by waterlogging. Early-waterlogging provoked great losses of root and shoot mass (plants attained 10 and 15% of controls,respectively) (**Table 3**). Late-waterlogging reduced these components to 29 and 31% of controls for roots and shoots, respectively (**Table 3**). Seed production was considerably reduced by both waterlogging treatments, where early- and late-waterlogged plants had only 4.4 and 9.5% of seed mass compared to controls (**Table 3**).”

The authors apologize for this error and state that this does not change the scientific conclusions of the article in any way. The original article has been updated.

