# Supplementary material for: Waterlogging of Winter Crops at Early and Late Stages: Impacts on Leaf Physiology, Growth and Yield
Source: Front Plant Sci. 2018 Dec 20;9:1863. doi: 10.3389/fpls.2018.01863 (PMC6306497; doi:10.3389/fpls.2018.01863)

Waterlogging of winter crops at early and late stages: impacts on leaf physiology, growth and yield

Ploschuk RA, Miralles DJ, Colmer TD, Ploschuk EL and Striker GG

Figure S1. (A) Air vapour pressure deficit (VPD_{air}, kPa), (B) maximum (T_{max}, ºC) and minimum (T_{min}, ºC) air temperature and (C) photoperiod (hours of daylight sunrise to sunset) during the experimental period expressed in days after sowing (DAS). Green-highlighted points in (A) denote the dates when physiological measurements (Fig. 1) were taken. Grey and black bars on the x-axis represent the 14-day early- and late-waterlogging periods, respectively.

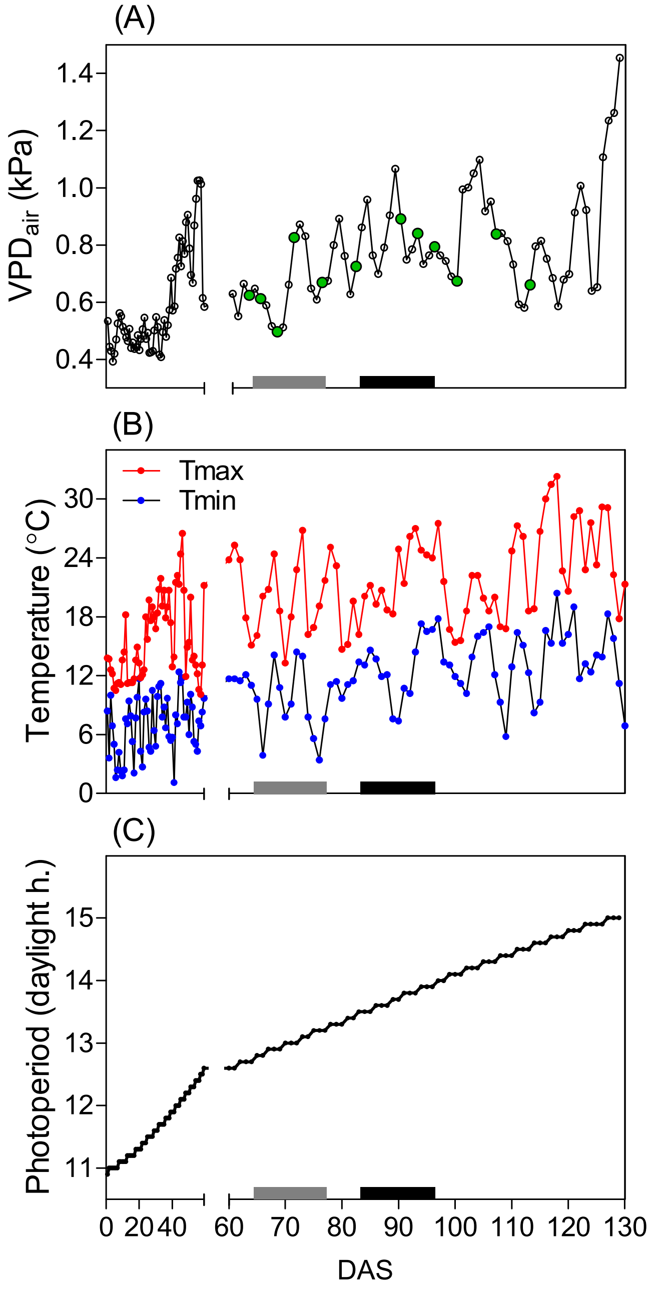

Figure S2. Soil redox potential at pH 7 (Eh_7) for control (well drained substrate) and waterlogged substrate (early- and late-waterlogging). Grey and black bars on the x-axis represent the 14-day period for early- and late-waterlogging, respectively. Values are means ± standard errors of 4 replicates.

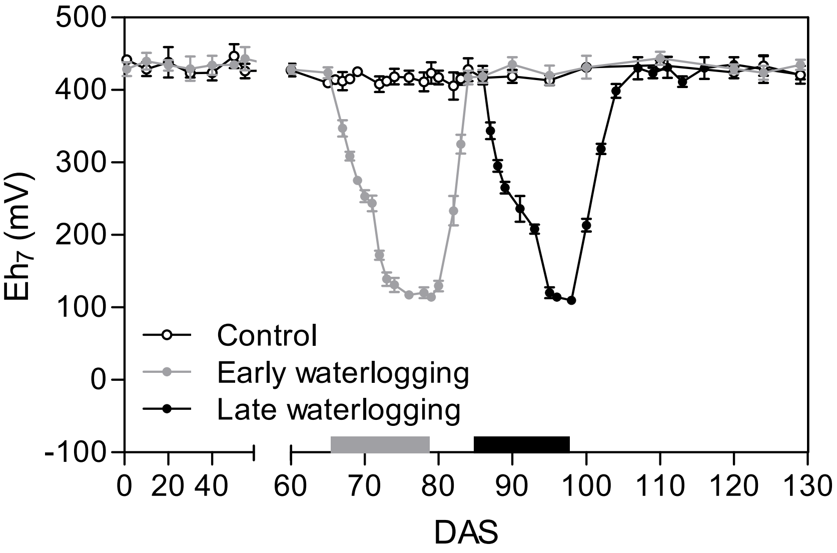

Figure S3. Flowering period of wheat, barley, rapeseed and field pea, represented by each horizontal line and expressed in days after sowing (DAS). As shown in the graph, rapeseed flowering period is more extended compared to the rest of the species. Arrows located on the right of the segments indicate the approximate beginning of seed filling for each species. Grey and black bars on the x-axis represent the 14-day period for early- and late-waterlogging, respectively.

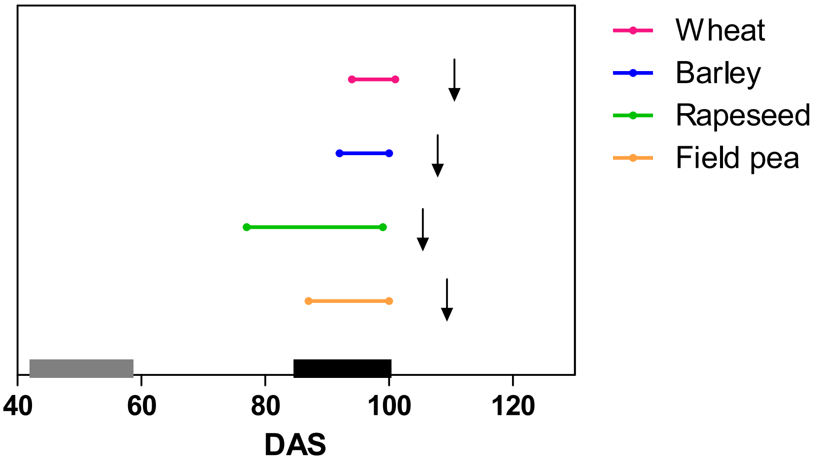

Supplement: Supplementary file 1 [file Data_Sheet_1.PDF]
